# Bladder sarcoma treated with radical cystectomy and adjuvant epirubicin and ifosfamide chemotherapy: a case report

**DOI:** 10.3389/fonc.2026.1838543

**Published:** 2026-05-12

**Authors:** Jiaxin Liu, Hui Han, Shuangping Lu, Siming Chen, Yuchen Jiang, Zhu Wang, Xuebing Han

**Affiliations:** 1 Department of Urology, Shanxi Province Cancer Hospital, Taiyuan, China; 2 Shanxi Hospital Affiliated to Cancer Hospital, Chinese Academy of Medical Sciences, Taiyuan, China; 3 Department of Urology, Cancer Hospital Affiliated to Shanxi Medical University, Taiyuan, China

**Keywords:** bladder sarcoma, case report, chemotherapy, magnetic resonance imaging, radical cystectomy

## Abstract

Bladder sarcoma is a rare mesenchymal tumor of the bladder, accounting for approximately 0.3% of all bladder tumors. Due to its low incidence and the lack of specificity in clinical symptoms and imaging findings, preoperative diagnosis is difficult. This article reports a case of a 53-year-old male patient who presented with painless gross hematuria. Ultrasound and computed tomography (CT) indicated a space-occupying lesion in the bladder. Cystoscopic biopsy indicated a malignant tumor, likely undifferentiated carcinoma or poorly differentiated sarcoma. The patient underwent laparoscopic radical cystectomy with pelvic lymph node dissection, ileal conduit, and appendectomy. Postoperative pathological examination, combined with immunohistochemical results, was considered to be a small round cell undifferentiated sarcoma. The patient received six cycles of adjuvant chemotherapy with epirubicin and ifosfamide (EI) after surgery. At the 12-month follow-up, the patient showed no evidence of tumor recurrence or distant metastasis. Currently, bladder sarcoma is clinically rare, and its pathogenesis remains poorly understood. Previous literature mostly consists of case reports, and there is a lack of unified treatment standards. The effectiveness of treatment methods still requires validation through more cases. This case report details the entire diagnosis and treatment process of a patient with bladder sarcoma, demonstrating the feasibility and short-term efficacy of radical surgery combined with the EI chemotherapy regimen, and provides a reference for the clinical diagnosis and treatment of such rare cases.

## 1 Introduction

Bladder cancer is one of the most common types of cancer worldwide (1). Among its various histological subtypes, urothelial carcinoma is the predominant pathological subtype, accounting for over 90% of all cases. In urothelial carcinoma, the 5-year progression-free survival rate for patients with non-muscle-invasive bladder cancer (NMIBC) can reach approximately 95%, while the 5-year overall survival rate for patients with localized muscle-invasive bladder cancer (MIBC) is about 55% (2). In contrast, primary bladder sarcoma (PBS) represents an extremely rare pathological subtype, originating from the mesenchymal tissue of the bladder and accounting for less than 1% of all bladder malignancies (3). Due to the low incidence of bladder sarcoma, there is a lack of large-scale clinical studies, standardized treatment guidelines, and established prognostic models. Clinical diagnosis and treatment primarily rely on case reports or small-sample retrospective studies. The morphology of bladder sarcoma is often atypical (4), making it prone to misdiagnosis as myofibroblastic tumor, sarcomatoid urothelial carcinoma, or metastatic sarcoma, thereby delaying diagnosis and treatment. Bladder sarcoma typically exhibits high aggressiveness and malignant biological behavior (5). Even with radical surgical treatment, the risks of local recurrence and distant metastasis remain high, leading to an overall poor prognosis.

For such high-risk patients, surgery alone is often insufficient to achieve long-term disease control. Postoperative systemic adjuvant therapy is regarded as a critical component in improving prognosis. For patients with positive margins, large tumor volume, or high histological grade, adjuvant therapy plays a significant role in treating micrometastases and delaying tumor recurrence (6). In the systemic treatment of soft tissue sarcoma, chemotherapy regimens based on anthracyclines combined with alkylating agents are widely used as first-line options. By synergistically interfering with tumor DNA synthesis and repair, this approach has been shown to significantly reduce the risk of postoperative recurrence and distant metastasis in certain high-risk patients with limb and trunk soft tissue sarcoma (7, 8). Currently, experience with the application of the epirubicin and ifosfamide (EI) regimen in patients with bladder sarcoma is extremely limited, and there is a lack of systematic evaluation regarding its efficacy, safety, and long-term prognosis.

Therefore, this article provides a detailed report on the diagnosis and treatment course of a patient with primary bladder sarcoma. The patient underwent radical cystectomy and completed six cycles of adjuvant chemotherapy with the EI regimen. The aim is to explore the application process, short-term efficacy, and safety of surgery combined with chemotherapy in patients with this rare and complex pathological type, to accumulate clinical experience and offer a valuable reference for clinical decision-making in similar cases.

## 2 Case description

**Figure 1:**
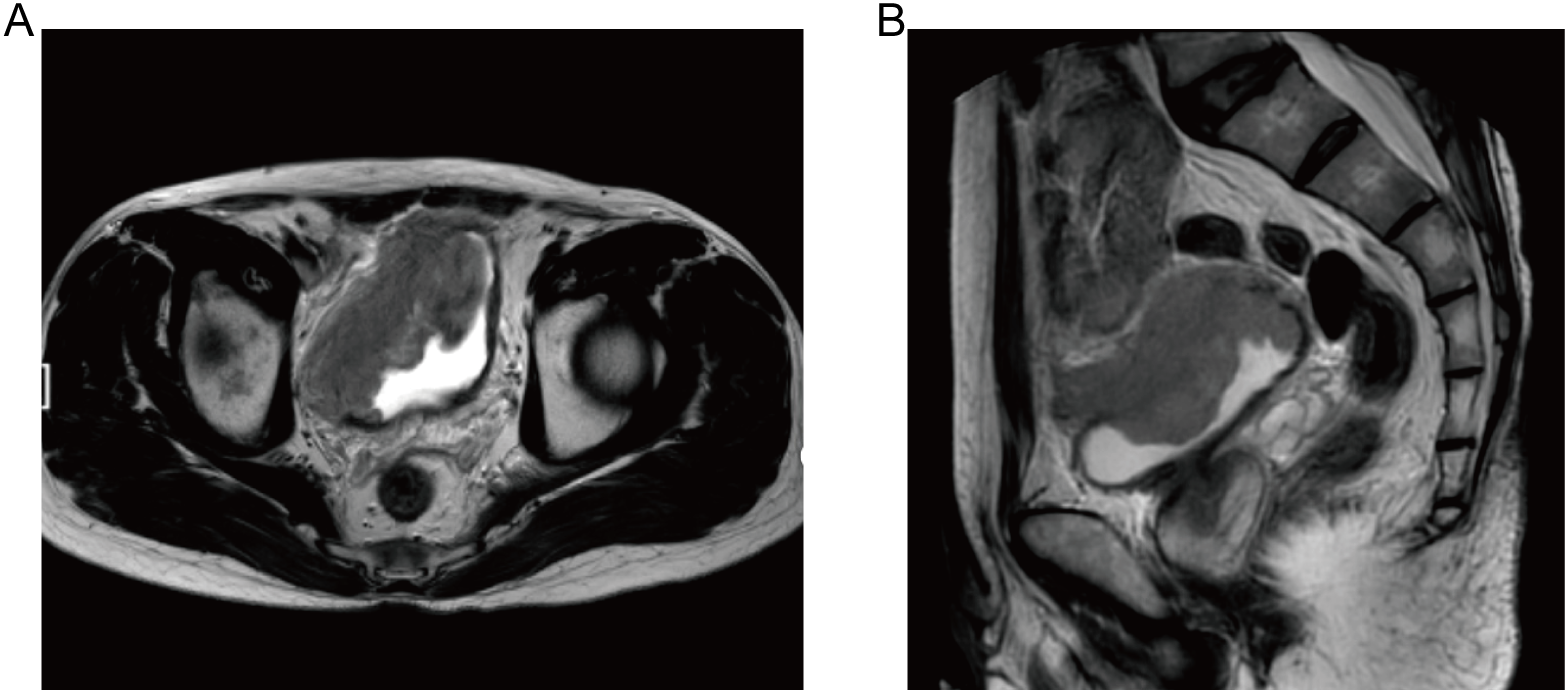
Pelvic MRI of the patient at diagnosis. **(A)** Axial T2-weighted imaging (T2WI) shows a space-occupying lesion in the right anterior and right lateral walls of the bladder with slightly high signal relative to the bladder wall. **(B)** Sagittal T2WI reveals a slightly high signal within the bladder compared with the bladder wall, with invasion of the perivesical fat space.

A 53-year-old male patient presented with painless gross hematuria present for seven days, accompanied by blood clots without any identifiable precipitating factors, and without associated urinary frequency or dysuria. The patient was treated in a local hospital. Urinary ultrasonography revealed a space-occupying lesion in the bladder. Computed tomography (CT) examination showed a solid mass on the right wall of the bladder, suspected to be malignant, with a clinical stage of T3bN0Mx. The patient was subsequently referred to our hospital for further treatment. The Patient denied any family history of hereditary cancer syndromes or tumor-related genetic diseases. His past medical history was significant only for prior surgical intervention for lumbar spondylolisthesis, with no other chronic comorbidities, history of infectious diseases, or relevant social or personal history. Upon admission, the patient underwent a series of comprehensive systemic examinations, including CT scans of the head, chest, abdomen, and pelvis, as well as pelvic magnetic resonance imaging (MRI). The results showed a bladder tumor with clinical stage T3bN0M0. The tumor had penetrated the bladder muscle layer and invaded the perivesical fatty tissue on the right side (**Figure 1**). Subsequently, the patient underwent cystoscopy for additional evaluation and biopsy. Cystoscopy revealed a broad-based, cauliflower-like mass measuring approximately 5 cm × 5 cm on the right lateral wall of the bladder, with abundant surface vascularity. Pathological examination of the biopsy specimen initially suggested a malignant tumor, favoring undifferentiated carcinoma or poorly differentiated sarcoma with neuroendocrine expression.

**Figure 2:**
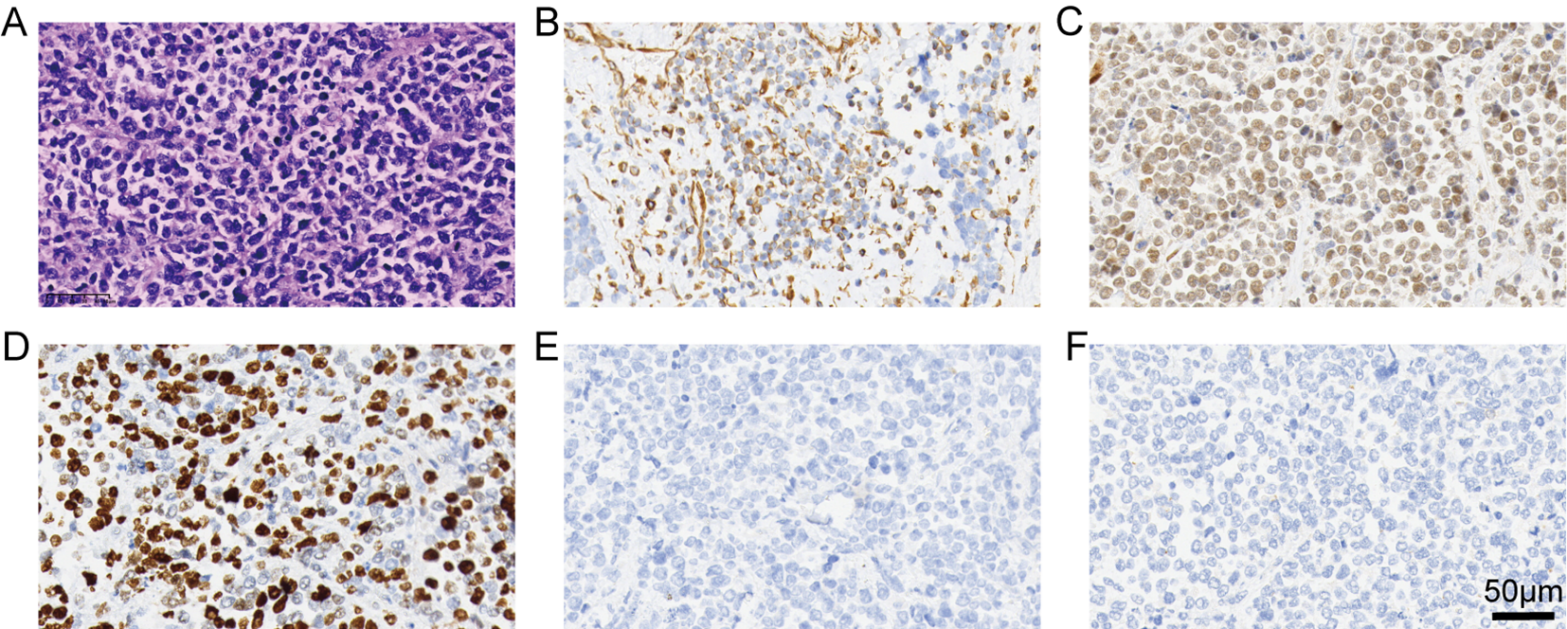
Pathological and immunohistochemical findings of the patient. **(A)** HE staining reveals no typical epithelial or glandular structures. The carcinoma cells are arranged predominantly in a diffuse and nested growth pattern with marked cellular atypia; the nuclei are enlarged with a high nuclear-cytoplasmic ratio, and mitotic figures are observed. **(B)** The tumor cells are diffusely strongly positive for vimentin. **(C)** The tumor cells are diffusely strongly positive for FLI-1. **(D)** The tumor cells are diffusely strongly positive for Ki-67. **(E)** Tumor cells show negative expression of GATA3. **(F)** Tumor cells show negative expression of AE1/AE3.

The patient was in excellent general condition and underwent laparoscopic radical cystectomy with pelvic lymph node dissection, ileal conduit, and appendectomy on March 25, 2025. Intraoperative frozen section analysis of the margins from both the right and left ureteral stumps revealed no evidence of carcinoma. Postoperative histopathological analysis diagnosed a small round cell undifferentiated sarcoma (**Figure 2**). Immunohistochemical staining results were as follows: Ki-67(+), MPO(-), SMARCA4(+), CD99(+), FLI-1(+), Vimentin(+), CK7(-), CK20(-), GATA3(-), Desmin (-), MyoD1(-), CD3(-), CD20(-), CD38(-), Syn(+), CgA(-), ERG(+), CD34(+), ALK(-), CD30(-), Melan-A(-), HMB45(-), S-100(-), and CD56(+), AE1/AE3(-). Based on the clinical, imaging, and pathological findings, the patient was ultimately diagnosed with small round cell undifferentiated sarcoma of the bladder.

**Figure 3:**
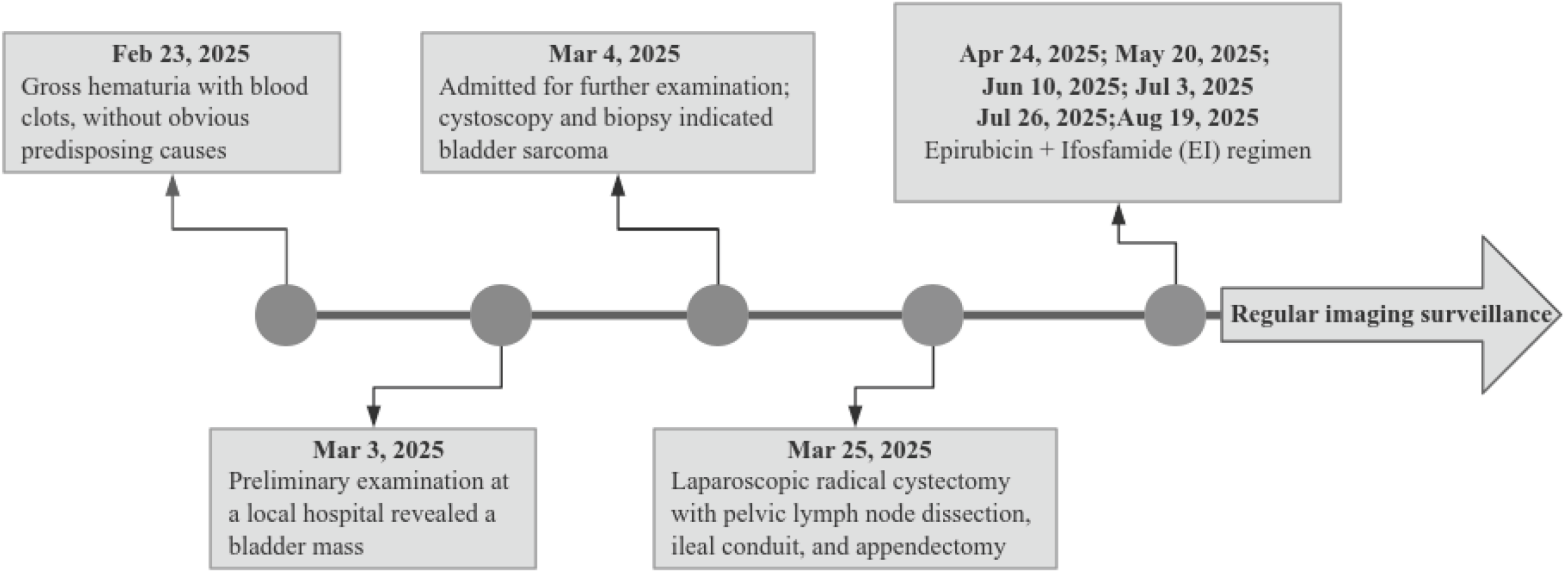
Patient clinical history. Includes the full process from initial evaluation, surgical management to subsequent postoperative follow-up.

Postoperatively, the patient received first-line chemotherapy with the EI regimen. Leukopenia occurred after the first cycle of chemotherapy, and the hemogram returned to normal after treatment with granulocyte colony-stimulating factor(G-CSF). Subsequently, the patient completed five additional cycles of this regimen without experiencing any significant adverse reactions. Regular follow-up imaging examinations performed at 6-month intervals, primarily consisting of chest and abdominal CT scans, have shown no evidence of tumor recurrence or metastasis (**Figure 3**).

## 3 Discussion

Bladder sarcoma is an extremely rare malignancy of the bladder, with an incidence accounting for less than 1% of all bladder malignancies (3). Because its clinical and imaging manifestations overlap with common urothelial carcinoma, it often leads to difficulties in preoperative diagnosis and a lack of standardized consensus on treatment options. Therefore, the diagnosis and treatment process of each confirmed patient is worth recording and discussing. Bladder sarcoma is characterized by high invasiveness, high malignancy, high metastasis rate and poor prognosis. Radical cystectomy combined with pelvic lymph node dissection is the main treatment method at present (9).

In this case, the patient underwent radical cystectomy. A multicenter analysis based on the National Cancer Database showed that the 5-year OS rate for patients with sarcomatoid variant who underwent radical cystectomy was 31.9%, significantly higher than the 23.3% in the bladder-sparing treatment group (P<0.001) (10). Similarly, for adult genitourinary sarcoma—a rare malignancy characterized by its unique anatomical location and highly heterogeneous biological behavior—complete surgical resection with attainment of negative margins is recognized as the core treatment principle (11). Multiple single-center and multi-institutional series have demonstrated that negative margin status is a key independent prognostic factor for reducing the risk of local recurrence, decreasing the occurrence of distant metastasis, and ultimately improving long-term survival (12). Studies have found that patients undergoing complete resection with negative margins have significantly lower local recurrence rates and improved 5-year disease-free and overall survival (13, 14). Furthermore, although evidence regarding the efficacy of pelvic lymph node dissection specifically for primary bladder sarcoma remains limited, in clinical practice for sarcomatoid urothelial carcinoma and soft tissue sarcoma, standard pelvic lymph node dissection has been incorporated into the surgical approach. It provides accurate pathological staging information and may offer potential survival benefits for certain patients at risk of lymphatic metastasis (15, 16).

Current research on bladder sarcoma remains at a relatively preliminary stage. In the treatment regimens for liposarcoma, synovial sarcoma, and leiomyosarcoma, chemotherapy plays a significant role, with the current first-line chemotherapy regimen being epirubicin plus ifosfamide (17, 18). Studies have shown that for certain advanced soft tissue sarcomas, targeted agents can serve as first-line treatment options; for example, anti-angiogenic tyrosine kinase inhibitors have been proven to extend progression-free survival in patients with advanced soft tissue sarcoma (19). High-dose ifosfamide is effective for bone and soft tissue sarcomas and can be used for advanced salvage therapy (20). A retrospective study showed that the objective remission rate of anthracyclines combined with ifosfamide in the first-line treatment of advanced myxofibrosarcoma was 31% (21). In this case, preoperative examinations revealed no tumor metastasis, and the preoperative pathological diagnosis was clear. Based on the disease assessment, the patient underwent radical cystectomy combined with pelvic lymph node dissection. Postoperative pathology further confirmed bladder sarcoma. The patient subsequently received adjuvant therapy with the combined EI chemotherapy regimen, completing six cycles of treatment as per protocol. During adjuvant chemotherapy, the patient presented with leukopenia, a manifestation of myelosuppression that is fully consistent with the well-characterized hematological toxicity profile of the EI regimen. Common adverse events of the EI regimen mainly include myelosuppression, cardiotoxicity, urinary tract toxicity, and neurotoxicity; in this case, only myelosuppression occurred in the initial phase of chemotherapy. This expected adverse event was promptly identified via regular hematological monitoring, and effectively managed with standardized supportive care using granulocyte G-CSF. No severe infection secondary to myelosuppression or treatment cycle delay occurred throughout the full treatment course, and the patient demonstrated good overall tolerance. As of the latest follow-up (12 months post-surgery), regular follow-up assessments indicate stable disease control, with no evidence of tumor recurrence or distant metastasis.

Based on existing clinical consensus and the experience from this case, the cornerstone of treatment for localized bladder sarcoma remains radical surgical resection. Postoperative adjuvant therapy, such as chemotherapy, as part of a comprehensive treatment approach, may further improve prognosis. This is particularly relevant for patients without tumor metastasis, who have a greater potential for achieving long-term disease control. However, due to the extreme rarity of bladder sarcoma cases, there are currently no unified diagnostic and treatment guidelines. Especially for patients with advanced distant metastasis, the efficacy and prognosis remain unsatisfactory, and effective treatment strategies are still lacking. Therefore, future efforts require the accumulation of more clinical cases and experiential summaries to further optimize clinical diagnosis and treatment protocols and improve patient survival rates.

## 4 Conclusion

In summary, radical cystectomy combined with the EI chemotherapy regimen achieved a favorable therapeutic effect in this patient with bladder sarcoma, with an acceptable safety profile. This report has several limitations, mainly including the single-case design and a relatively short follow-up period of 12 months. If the efficacy and safety of this therapeutic strategy can be replicated in larger cohorts, prospective studies may first be conducted to explore the potential role of surgery combined with chemotherapy in the management of bladder sarcoma. Subsequently, comparative trials could be performed to determine the optimal duration of chemotherapy, such as comparing fixed-duration versus prolonged regimens.

## 5 Patient perspective

I was diagnosed with bladder sarcoma after suffering from dysuria, frequent urination and gross hematuria. Under the professional treatment of the medical team, I received radical surgical resection followed by an adjuvant EI chemotherapy regimen. To date, my urinary symptoms have been significantly relieved, and I am still under regular follow-up as scheduled. I am very grateful for the meticulous care provided by the medical team, which has helped me return to a normal life and maintain a positive attitude in fighting the disease.

## Data Availability

The original contributions presented in the study are included in the article/supplementary material. Further inquiries can be directed to the corresponding authors.
